# Case Report: Intact Survival of a Marginally Viable Male Infant Born Weighing 268 Grams at 24 Weeks Gestation

**DOI:** 10.3389/fped.2020.628362

**Published:** 2021-02-03

**Authors:** Takeshi Arimitsu, Daiki Wakabayashi, Satoshi Tamaoka, Mona Takahashi, Mariko Hida, Takao Takahashi

**Affiliations:** Department of Pediatrics, Keio University School of Medicine, Tokyo, Japan

**Keywords:** intact survival, extremely low birth weight, male, birth weight of 268 g, marginally viable infant, minimal handling, family involvement

## Abstract

We report the case of a preterm small for gestational age male infant born at 24 weeks of gestation with a birth weight of 268 g who was discharged from our hospital without the requirement for home oxygen therapy or tube feeding. He did not experience severe intraventricular hemorrhage, periventricular leukomalacia, hearing disability, or any other serious complications. At that time (February 2019), according to the University of Iowa's Tiniest Babies Registry, he was the tiniest male infant in the world to survive without any serious complications other than severe retinopathy of prematurity that required laser therapy. Although the survival rate of infants with extremely low birth weight is improving worldwide, a high mortality rate and incidence of severe complications remain common for infants weighing <300 g at birth, particularly in male infants. In recent years, there have been frequent discussions regarding the ethical and social issues involved in treating extremely preterm infants weighing <400 g. Despite the challenges, reports of such infants surviving are increasing. Neonatal medicine has already achieved great success in treating infants weighing 400 g or more at birth. However, lack of evidence and experience may make physicians reluctant to treat infants weighing less than this. The present case demonstrates that intact survival of a marginally viable male infant with a birth weight of <300 g is possible with minimal handling and family involvement beginning shortly after birth. Our detailed description of the clinical course of this case should provide invaluable information to physicians around the world who treat such infants. This report will aid in the progress of neonatal medicine and help to address many of the social and ethical issues surrounding their care.

## Introduction

Detailed case reports of infants with a birth weight of <300 g are extremely limited because of their exceedingly low survival rate (1–9). In particular clinical outcomes are extremely unfavorable for male infants in this category (1, 3, 10, 11). As of February 2019, only 23 infants weighing <300 g at birth were registered in the University of Iowa's Tiniest Babies Registry, of whom only four were male (12). However, a report of the successful discharge of an infant born in 1938 with a birth weight of 283 g indicates that some of such infants are viable with appropriate treatment (12). The ongoing worldwide improvement in the survival rate of infants with extremely low birth weight can be attributed to physicians' accumulation of experience and evidence over the years. Continuing accumulation of case reports is essential to improve clinical outcomes for some of such infants.

Neonatal medicine has already achieved great success in caring for infants weighing 400 g or more at birth (1, 3). However, physicians may hesitate to treat infants born weighing less than this, particularly those below 300 g, because of the unfavorable prognosis and lack of evidence-based practice. For male infants in particular, a high mortality rate and incidence of severe complications are still unresolved issues (10, 11). However, there have been several recent reports of survival of infants weighing <300 g at birth (1, 8, 9). There is currently an active discussion regarding the appropriate ethical and social approach to treat infants with extremely low birth weight.

We report the case of a preterm small for gestational age male infant who was born at 24 weeks of gestation with a birth weight of 268 g and was discharged from our hospital without requiring home oxygen therapy or tube feeding. He did not experience severe intraventricular hemorrhage, periventricular leukomalacia, hearing disability, or any other serious complications. This case illustrates the importance of both minimal handling and family involvement.

The concept of minimal handling has been familiar to pediatricians since 1980 (13–18). Today, this has become a standard of care, especially in preterm infants, as it promotes cardiopulmonary stability and is associated with good long-term outcomes. However, it is unclear to what degree minimal handling standards can be maintained with marginally viable premature infants, as handling may be necessary for evaluation and treatment. In addition, evidence regarding the application of minimal handling in these infants is limited, as there are few reports on survivors in this population, and those that exist have generally involved infants weighing more than 300 g at birth. Thus, our report provides information on the application of minimal handling standards for marginally viable premature infants.

Family involvement is the standard of care for premature infants admitted to the newborn intensive care unit (NICU) and can be regarded as the essential supporting foundation of NICU treatment. It strengthens bonds among family members, reduces stress for both the babies and their families, and promotes infant stability and development (19–21). Despite these benefits, hospital staff often hesitate to involve the family in the care of marginally viable premature infants because of the risk of infection and the infants' instability, and there are few reports regarding the effects of family involvement in these infants. We consider family involvement especially important in these cases because the stress placed on the families is extraordinary. We hope our report of successfully involving the family in the treatment of a vulnerable premature infant will encourage hospital staff to increase family involvement in these cases.

## Case Description

**Figure 1:**
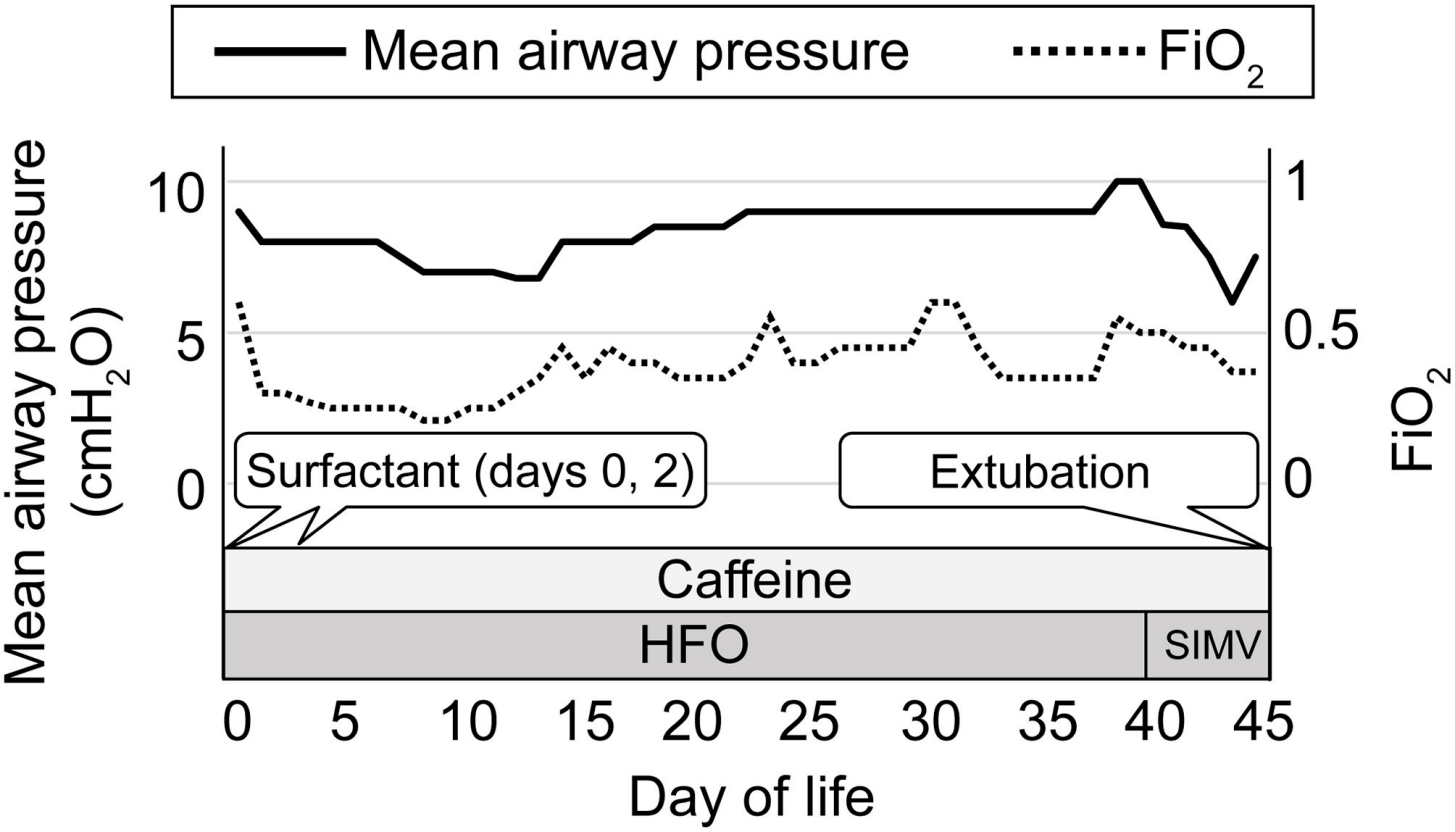
The clinical course of respiratory management. The left vertical axis indicates the daily maximum ventilator setting for mean airway pressure and the right vertical axis indicates the daily maximum ventilator setting for FiO_2_. The horizontal axis indicates the postnatal day of life. The solid line indicates mean airway pressure, and the dotted line indicates FiO_2_. HFO, high-frequency oscillation; SIMV, synchronized intermittent mandatory ventilation.

A male infant was born to a 30-years-old mother with no significant medical history (gravida 0, para 0, abortus 0) who became pregnant *via* natural insemination. At 23 weeks of gestation, the mother was referred to our hospital because of severe fetal growth restriction caused by utero-placental insufficiency and was treated with antenatal steroids. Fetal maturity was ascertained by calculating from the mother's last menstrual period and was confirmed by prenatal ultrasound. Prenatal counseling was conducted for the parents, and they requested to give birth by cesarean section. At 24 weeks and 0 days of gestation, the infant was delivered by emergency cesarean section because of interrupted blood flow in the umbilical cord; there was no umbilical cord coiling or amniotic fluid staining. The weight of the placenta was 60 g. Placental pathology revealed infarction. No findings of chorioamnionitis were observed. Immediately after birth, he was wrapped using a plastic bag on a prewarmed radiant warmer. The ambient temperature of the operating room was set to 27°C (22). He was intubated in the operation room with a 2-mm endotracheal tube and transferred to the NICU in an incubator. The admission temperature at the axilla was 35.8°C. The humidity in the incubator was set at 90% on day 0 in order to reduce insensible water loss, and it was lowered by 5% daily to 60% (23). His Apgar score was 2 at 1 min after birth, 6 at 5 min, and 6 at 10 min. Apart from his size, his physical examination and laboratory test results were unremarkable. Postnatal maturation assessment was adequate for a preterm infant born at 24 weeks of gestation. Genetic disorders were ruled out as a cause of intrauterine growth restriction based on the findings of physical examination and ultrasonography and the results of blood examination including blood gas, complete blood count, and biochemistry. Hence, genetic testing was not performed. His birth weight was 268 g (−3.92 SD). In order to prioritize stabilization, his head circumference and length were not measured at birth; when they were measured for the first time on postnatal day 16 (postmenstrual age 26 weeks, 2 days), his head circumference was 18.5 cm (−3.03 SD) and his length was 24.0 cm (−3.7 SD). On arrival to the NICU, he was placed on high-frequency oscillatory ventilation because of low pulmonary compliance, and a surfactant was administered on days 0 and 2. Sedation was not performed. Anhydrous caffeine was started from day 0. Prophylactic antibiotics (ampicillin, gentamicin, and fosfluconazole) were administered after birth and continued until day 6. Because the umbilical artery was narrow, an umbilical artery catheter could not be inserted. The infant's blood pressure was measured after birth *via* a 24G peripheral arterial catheter to avoid the risk of damage that a cuff would pose to his immature skin and was the approximate value expected for his gestational age; thus, no inotropes were administered. After the catheter occluded on day 8, we assessed his blood pressure mainly by physical examination. The target ranges of osmotic agents were set with reference to previous studies, and his albumin level was kept above 2.5 g/dL to maintain osmotic pressure (24–27). Echocardiography was performed to diagnose patent ductus arteriosus (PDA) on day 1. We administered 0.1 mg/kg per dose of indomethacin, because echocardiography represented an increased shunt volume of PDA (28, 29). PDA closed after indomethacin administration on day 1. He required 45 days of mechanical ventilation, including 40 days of high-frequency oscillation (Figure 1). He was extubated on day 45 (30 weeks and 3 days of postmenstrual day) when we confirmed that the prongs of our continuous positive airway pressure machine fit his nose. His body weight when measured after extubation was 518 g on day 51 (31 weeks and 2 days of postmenstrual day). From day 42, he received a systemic steroid (dexamethasone) for 10 days, as advised by Doyle et al. (30). After successful extubation, he required nasal continuous positive airway pressure for 7 days and a high-flow nasal cannula for 116 days. Target SpO_2_ was set at 85–95% before 34 weeks of postmenstrual age and above 95% afterward.

Parenteral nutrition was started on day 0 *via* an 18G umbilical venous catheter. After this catheter became occluded on day 12, a 27G peripherally inserted central venous catheter was used, but it also became occluded on day 19. From then on, parenteral nutrition was continued *via* a peripheral venous catheter. On day 1, oral administration of the mother's breast milk using a cotton swab was performed by the parents, and trophic feeding *via* nasogastric tube was started. A maximum of 3 mL/kg of 5-fold-diluted amidotrizoic acid was rectally administered for 1 week starting on day 1, resulting in successful defecation. After 3 days of trophic feeds, enteral feeding was stopped because the infant's abdomen was gradually becoming distended. His daily abdominal X-ray indicated significantly expanding intestinal gas, and we diagnosed him with meconium-related ileus. Intragastric administration of 3 mL/kg 5-fold-diluted amidotrizoic acid succeeded in triggering mass excretion of feces, as described in previous studies (31–35). Subsequently, abdominal distention improved, and enteral feeding was resumed. Fortified breast milk was used from day 20 when the infant's breast milk consumption reached 100 mL/kg per day. We maintained him on a breast milk diet for most of his hospital stay, adding medium-chain triglyceride oil from day 94 to 133 for calorie enrichment. The calorie goal was set at 120–140 kcal/kg/day. Oral feeding was initiated on day 98, and the gastric tube was removed on day 137. During the treatment, blood glucose was managed within the reference range with glucose administration and enteral feeding only, except for insulin administration at day 1 and from day 5 to 6. Fluid intake was determined from vital signs and physical findings, fluid balance, and blood test results. Fluid intake was relatively restricted in expectation of reducing risk of morbidities and complications (36). Transepidermal water loss was estimated based on the sodium levels, fluid intake, urine output, and previous reports (37). Total fluid volume administered on day 0 was 65 mL/kg; for most of the period after the patient stabilized, it was maintained between 130 and 160 mL/kg per day. Minimal handling was conducted in all aspects of the patient's management. As described above, anthropometric measurements were kept to a minimum, and our patient's weight was estimated from fluids and nutritional balance and was adjusted on the day it was measured. After his first weighing on day 0, he was not weighed again until day 30 to confirm the anticipated growth. His body weight, length, and head circumference were measured every 2 weeks after that. Until day 42, we kept him in a prone position without postural change, aiming to prevent both gastroesophageal reflux and unplanned extubation. During that period, we undertook careful skin management, making sure to give him proper support to prevent bedsores. Although minimizing postural change may increase the risk of skin and pulmonary infection, there were only two events after the day of birth when we had to administer antibiotics: from day 38 to 45 for suspected aspiration pneumonia and from day 66 to 76 for cellulitis due to methicillin-sensitive *Staphylococcus aureus* at the site of the removed peripheral catheter. Blood sampling was also minimized to reduce the frequency of painful procedures and the risk of iatrogenic anemia. For a month after birth, heel sticks were performed as little as once per day to analyze blood gas, electrolytes, metabolic factors (glucose, lactate), and biochemical factors (albumin, phosphate, C-reactive protein). We performed a comprehensive blood examination (complete blood count and biochemical tests) only once every week or two (Table 1).

**Table 1:** The average number of daily heel sticks and endotracheal suctions for NICU patients during the first 2 weeks of life.

**Source**	**Heel sticks**	**Endotracheal suctions**
Simons et al. (38)	1	3.3
Carbajal et al. (39)	3.2	3.7
Cignacco et al. (40)	1.2	1.9
Jeong et al. (41)	1	1.6
Guedj et al. (42)	1.4	2.4
Britto et al. (43)	2.4	0.5
Roofthooft et al. (44)	1	3.3
Present case	3	4.7

*We calculated the average number of daily procedures per patient. NICU, newborn intensive care unit*.

In addition to minimal handling, we encouraged parental involvement from the day of birth. Shortly after the infant was born, the family began to talk to him and hold him. The parents frequently visited our NICU to engage in physical contact with him, including breastfeeding, which was performed from day 1 (Supplementary Video 1).

**Figure 2:**
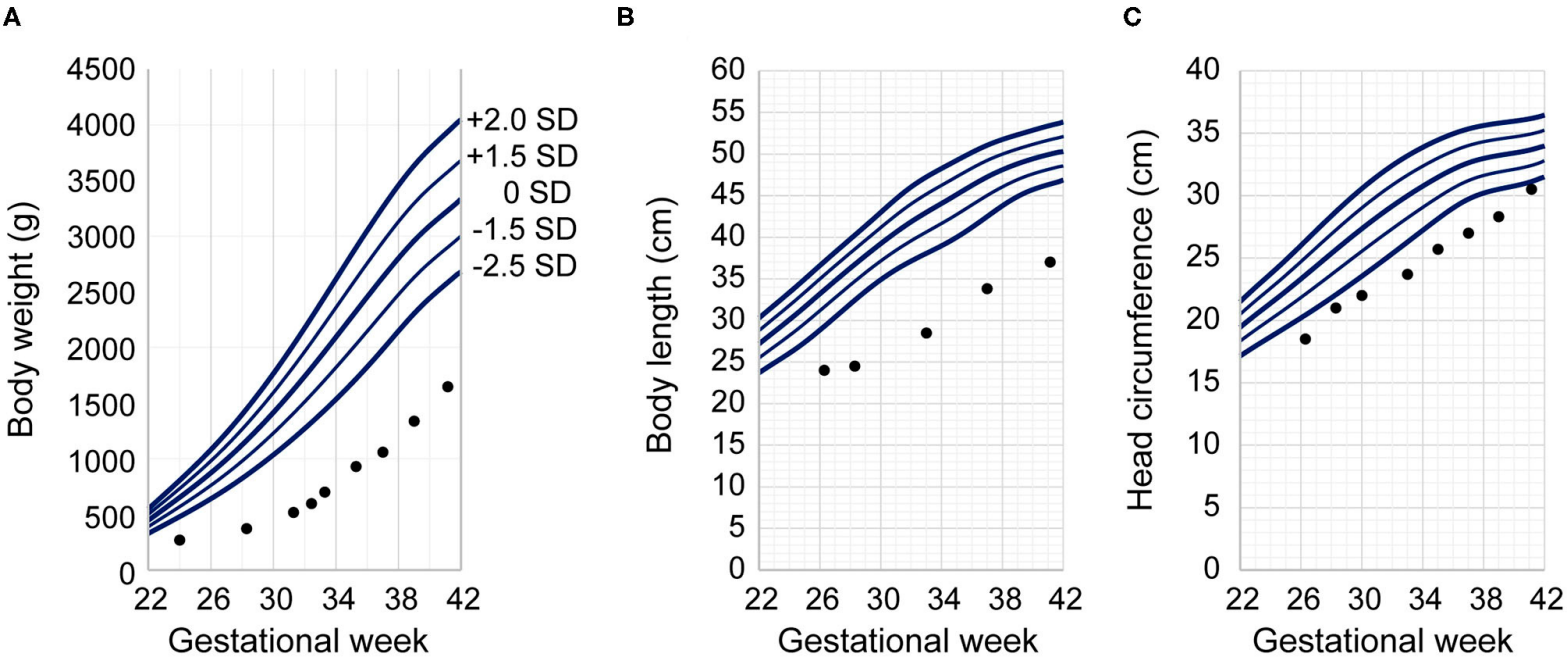
Patient's growth charts. Our infant's anthropometric measurements are plotted against Japanese neonatal anthropometric charts of male infants delivered by a para 0 mother according to gestational age at birth. Dots indicate the patient's measurements at the given postmenstrual age. The vertical axes indicate **(A)** body weight, **(B)** body length, and **(C)** head circumference. The horizontal axis indicates gestational age for the Japanese neonatal anthropometric charts and postmenstrual age for our patient. The Japanese neonatal anthropometric charts feature five solid lines indicating the distribution of data; in order from top to bottom, they represent the mean +2.0 SD, +1.5 SD, ±0 SD, −1.5 SD, and −2.0 SD.

Magnetic resonance imaging of the head at 45 weeks of postmenstrual age revealed no detectable abnormalities. An automated auditory brainstem response test was bilaterally passed. However, the infant developed bilateral retinopathy of prematurity (stages 2–3), requiring laser treatment. He was discharged from the hospital on day 176 (postmenstrual age, 49 weeks and 1 day) without any requirement for home oxygen therapy or tube feeding. His body weight at discharge was 3,238 g (−3.41 SD), his head circumference was 35.5 cm (−1.31 SD), and his length was 46.8 cm (−4.55 SD) (Figure 2) (45). The patient's mother said that she could only say how happy she was that he had grown so much.

## Discussion

Although neonatal medicine has advanced enough to produce favorable clinical outcomes for most premature infants, physicians still hesitate to treat those weighing <300 g at birth, especially male infants, because of lack of experience and evidence (1–12). Case reports of male infants weighing <300 g at birth are especially rare owing to a survival rate far lower than that of the female infants of similar weight. The present case report recounts the detailed clinical course of a small for gestational age male infant weighing 268 g at 24 weeks gestation; as of February 2019, he was the tiniest male infant in the world to be discharged from the hospital without any serious complications other than severe retinopathy of prematurity that required laser therapy (12, 46).

Antenatal steroid treatment contributed greatly to the absence of serious complications in the present case (47). The absence of preterm pre-labor rupture of membranes, which causes chorioamnionitis and oligohydramnios, was also beneficial for the survival of the infant (48, 49). Although the body weight at birth of the present case was extremely low, the successful clinical course was also related to the fact that the child was born at 24 weeks of gestation due to intrauterine growth restriction instead at 22 or 23 weeks of gestation (50). The primary cause for the severe retinopathy of prematurity was likely to be short gestational age and extremely low birth weight (38, 39). Bronchopulmonary dysplasia might also increase its risk (40). Further research is needed to improve the outcomes for marginally viable infants.

Table 1 shows that the number of heel sticks and endotracheal suctions during the first 2 weeks of life in our case is similar to that reported in previous cases (41–44, 51–53). During this period, our patient underwent three heel sticks (range 1–5), and 4.7 endotracheal suctions (range 1–9) per day on average; on some days, he received as few as 1 of each. Surprisingly, these numbers were similar to those reported in previous cases involving infants with a birth weight of more than 300 g. If handling can be safely minimized, this can improve patient stability and promote development (13–18). However, the necessary degree of handling should be determined by the physician, as minimal handling also means less information on the patient's situation. Fortunately, our hospital has previous experience with infants with a birth weight of <300 g (8), which helped us minimize handling in the present case.

Regarding family involvement during the acute phase of treatment, concerns about infection and instability may lead some physicians to refrain from encouraging families to proactively seek contact with their infants. However, we believe that encouraging families to hold their infants and talk to them contributes to infant stability and improves long-term development (19–21). The present case demonstrates that family involvement beginning soon after birth can be achieved safely and can positively affect the family's emotional well-being and the infant's clinical outcomes.

A limitation of this report is that the number of infants weighing <300 g at birth who could survive is unclear. The University of Iowa's worldwide database of surviving infants with birth weights <400 g includes cases reported in media outlets and medical journals, as well as reports submitted directly by physicians, but it is possible that some of those cases may not be included (12). However, the database mentioned above is currently the largest database of surviving marginally viable premature infants worldwide. Given the low survival rates reported in the literature, it is likely that the actual number of survivors in this population is extremely low (1–11).

Our experience, in this case, demonstrates that minimal handling can be safe and effective in treating infants with birth weight <300 g and that family interaction beginning soon after birth can positively affect clinical outcomes and family relationships with no adverse effects. We conclude that improved clinical outcomes and strong family relationships can be achieved for marginally viable premature infants through meticulous consideration of minimal handling and family involvement together with the application of up-to-date research and advanced medical devices. This report describes a very rare case that could be a source of invaluable information for the development of neonatal medicine. Further research developments are needed.

## Data Availability Statement

The original contributions presented in the study are included in the article/Supplementary Material, further inquiries can be directed to the corresponding author/s.

## Ethics Statement

Ethical review and approval was not required for the study on human participants in accordance with the local legislation and institutional requirements. The patients/participants provided their written informed consent to participate in this study. Written informed consent was obtained from the participants' parents for the publication of this case report and any identifiable data or images.

## Author Contributions

TA was an attending physician of the patient, performed the literature review, and wrote the first draft of the manuscript. DW, ST, and MT assisted in the patient's treatment. MH and TT supervised the patient's treatment, provided scientific contributions, and critically revised the paper. All authors read and approved the final version of the manuscript.

## Conflict of Interest

The authors declare that the research was conducted in the absence of any commercial or financial relationships that could be construed as a potential conflict of interest.
