# Supplementary material for: Case Report: Intact Survival of a Marginally Viable Male Infant Born Weighing 268 Grams at 24 Weeks Gestation
Source: Front Pediatr. 2021 Feb 3;8:628362. doi: 10.3389/fped.2020.628362 (PMC7888275; doi:10.3389/fped.2020.628362)
Supplement: Supplementary file 1 [file Table_1.DOCX]

Supplementary Material

**
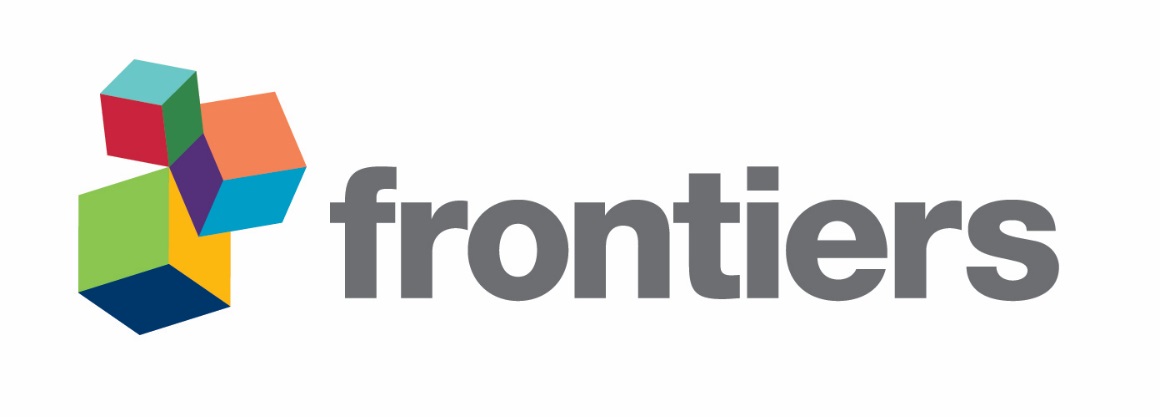
**

**Supplementary Video 1.** Oral breast milk administration by a parent.

The video shows breast milk administration being performed by one of the parents on postnatal day 28. In the video, the parents have the following conversation in Japanese: “Do you still want it?” “He is sucking it, right?” “Yeah, he is sucking it, right?” “Yeah. It’s cute.”
